# Rural/Urban Differences among American Indian/Alaska Native Peoples in Health Care Access and Outcomes, 2019–2023

**DOI:** 10.1007/s10900-025-01538-7

**Published:** 2025-11-13

**Authors:** Katy Backes Kozhimannil, Hailey A. Baker, Julia D. Interrante, Kyle X. Hill, Melissa L. Walls

**Affiliations:** 1 Division of Health Policy and Management, University of Minnesota School of Public Health, Minneapolis, USA; 2 Division of Environmental Health Sciences, University of Minnesota School of Public Health, Minneapolis, USA; 3 Department of International Health, Johns Hopkins University Bloomberg School of Public Health, Baltimore, USA

## Abstract

Health challenges affecting American Indian/Alaska Native (AI/AN) individuals are well-documented, but rural/urban differences remain understudied. This analysis describes health and health care access for rural and urban AI/AN adults, with attention to the role of the Indian Health Service (IHS), which primarily serves rural areas. Using 2019–2023 National Health Interview Surveys, we included adult respondents identifying as AI/AN, regardless of other reported races and ethnicities. County of residence was classified as urban (metropolitan) or rural (non-metropolitan). Outcomes included usual place of care, cost-related underuse of care, emergency room visits, clinic visits, worries about paying medical bills, and self-rated health. We found that AI/AN rural residents had greater socio-economic risks (lower education and higher poverty levels), but more access to IHS than urban AI/AN adults (*p <* .01, all comparisons). Rural-residing AI/AN individuals were more likely than urban AI/AN people to have a usual place of care (93.8% vs. 84.8%, *p* < .01) and to avoid cost-related underuse of care (75.9% vs. 65.3%, *p* < .01). Nearly 3 in 4 AI/AN adults –rural and urban - described their health as excellent, very good, or good; however, this was higher for urban (78.5%) vs. rural (73.8%) respondents (*p =.*015). Rural and urban AI/AN adults with IHS were more likely to have a usual place of care and lower cost barriers than those without IHS (*p* <.01, all comparisons). Socio-economic risks are higher for rural vs. urban AI/AN adults, but care was more accessible and affordable among rural AI/AN adults, who had comparatively greater access to IHS care.

## Background

There are 574 federally recognized Native nations in the United States (U.S.), each with its own unique culture, language, and history [1]. American Indian and Alaska Native (AI/AN) individuals who are citizens and descendants of these nations reside in all 50 states and the District of Columbia, living in both rural and urban areas [2]. While health care disparities affecting AI/AN individuals are well-documented, AI/AN health is often assessed in comparison to other populations, such as the non-Hispanic white demographic [3, 4]. However, limited research exists examining the rural/urban differences among AI/AN individuals, thus restricting our understanding of the health care experiences and needs for AI/AN people in the U.S.

Notably, an estimated 40% of AI/AN individuals live in rural areas, a higher proportion than any other racial or ethnic group in the U.S. [2] All people living in rural U.S. communities face greater access barriers and higher mortality rates than their urban counterparts, potentially contributing to the elevated disease burden experienced by AI/AN communities [5]. For example, the COVID-19 pandemic highlighted increased health risks experienced by rural and AI/AN people, who were more likely to experience hospitalization or death from the virus than all other racial and ethnic groups, [6] and both AI/AN and rural residents who were pregnant experienced a sharply increased risk of maternal mortality during the pandemic [7]. Poor health outcomes in both AI/AN and rural communities have long been influenced by broad challenges, including inadequate healthcare infrastructure, financial constraints, and geographic isolation [1, 8]. For Indigenous Peoples, these challenges also include long-term impacts of colonization [9]. 

Codified in perpetuity under the Indian Health Care Improvement Act when it was signed into law as part of the Affordable Care Act, [10] the United States has a trust responsibility to provide health care for AI/AN individuals. It does so through the provision of health care services at three types of facilities: Indian Health Service (IHS), Tribal, and Urban facilities, collectively referred to as the ITU system. Each facility type differs in funding, management, and intended patient population. IHS facilities are entirely federally funded and operated, while Tribal facilities are tribally operated with partial funding from the IHS. These IHS and Tribal facilities are primarily located in rural areas. Urban facilities, on the other hand, are funded through a variety of local, state, and federal sources as well as the IHS – however, only 1% of IHS funding is directed towards these facilities despite the majority of AI/AN individuals residing in urban areas [11]. This variation in operation and funding of these facilities are relevant when examining health among different groups of people within AI/AN communities, specifically as it relates to access to health care for rural and urban AI/AN individuals [3]. The purpose of this project is to describe rural/urban differences in health care access and outcomes for AI/AN adults in the U.S. from 2019 to 2023, with attention to access to the role of the IHS.

## Methods

We used pooled, cross-sectional data from the 2019 through 2023 National Center for Health Statistics, National Health Interview Survey (NHIS), [12] focusing on all adult respondents who self-identified as AI/AN, including those who reported AI/AN alone or AI/AN in combination with any other racial or ethnic group [13]. In the NHIS, AI/AN identity is a selection option in a question on racial identification, but citizenship in a Native nation is a political designation, [14] which is not captured in this survey. NHIS data do not contain information on Tribal enrollment or affiliation, or on if AI/AN respondents lived on reservations. Still, it is essential to analyze available data on AI/AN health, acknowledge limitations, and improve data collection and stewardship to illuminate health challenges faced by Indigenous Peoples; failure to do so contributes to demographic erasure [15, 16]. 

### Measurement

We examined key measures of health care access and outcomes, including the following: (1) whether the respondent reported no cost-related underuse of care in the past year (with cost-related underuse of care defined as either delayed care due to cost or being unable to afford needed care, including medical, dental, mental health, or prescription medications), (2) whether the respondent reported being not worried about paying medical bills (not worried vs. very worried/somewhat worried), (3) whether the respondent reported a usual place of medical care, (4) whether the respondent reported one or zero emergency visits in the past year, including either emergency room (ER) or emergency department (ED) visits, (5) whether the respondent reported less than 3 years’ time since they last had a clinic visit (either a doctor’s visit or a wellness visit), and (6) whether the respondent reported excellent, very good, or good self-rated health (vs. fair or poor health).

Key variables of interest examined for within-AI/AN differences were rurality, health insurance, and IHS access. Rurality was defined by the county of residence of the respondent and categorized as urban (metropolitan) or rural (non-metropolitan) based on the National Center for Health Statistics Urban-Rural Classification Scheme [17]. While the IHS is a health care delivery system and not health insurance, access to IHS care was assessed through the multiple-choice NHIS survey question: “What kinds of health insurance or health care coverage do you have?” We followed National Center for Health Statistics guidance and research precedent for measurement of insurance variables in this analysis, [12, 18, 19] and categorized health insurance as (1) private insurance only, (2) private and public insurance, (3) public insurance only (Medicare, Medicaid, Military related health care including TRICARE (CHAMPUS)/VA health care/CHAMPVA), and (4) uninsured (No coverage of any type or don’t know). We split these four health insurance categories based on whether respondents reported access to the IHS, leading to eight total insurance categories: each of the above listed categories, stratified by whether the respondent reported IHS care.

We also included sociodemographic characteristics known to be associated with differences in health care access and outcomes, [20] including sex (female or male), age (18–44, 45–54, 55–64, or 65+), education (less than high school, high school degree/equivalent, some college/Associate’s degree, Bachelor’s degree or more), disability status (yes/no, defined using the Washington Group Short Set Composite Disability Indicator), [21, 22] marital status (married or not married), and household income as a ratio to the federal poverty level (< 1.0, 1.0–1.99.0.99, 2.0–2.99.0.99, 3.0–3.99.0.99, or 4.0+).

## Analysis

We conducted descriptive analyses of key outcome measures described above between rural/urban adult AI/AN respondents. We conducted additional descriptive analyses among adult AI/AN respondents examining within-rural and within-urban differences by access to the IHS. We used χ^2^ tests to determine statistical significance, reporting *p*-values with a significance threshold of : *p* <.05. Data were pooled and weighted in accordance with NHIS recommendations, [23] and all analyses used NHIS provided survey weights to account for complex survey design, nonresponse bias, and multiple survey cycles. Missing data were limited but not negligible; 202 respondents (7.8%) were missing information for at least one sociodemographic covariate or outcome. In the main analysis, all respondents were included regardless of missing values; missing values were included in referent categories for each dichotomous (0/1) variable. We performed two sensitivity analyses regarding missing data: (1) excluding those missing any sociodemographic characteristics (*n* = 144), and (2) excluding those missing any sociodemographic characteristics or outcomes (*n* = 202). Results were largely consistent, with the only difference arising under a complete case analysis where differences in emergency care based on IHS access became statistically significant at *p* <.05 (data not shown). All analyses were conducted using SAS, version 9.4 (Cary, NC).

This analysis was reviewed and determined “not human subjects research” by the University of Minnesota, Institutional Review Board. All core members of the study team completed the Tribal-University Relations training and reviewed the Indigenous Research Guidebook. These resources, prepared by University of Minnesota, are designed to guide research involving work with Tribal collaborators, Tribal communities, Tribal natural resources, and other Tribally controlled or Tribal-serving institutions, Indigenous Peoples, places, and objects of cultural significance.

## Results

This study included data from 828 rural and 1,766 urban AI/AN residents. Table 1 describes differences in sociodemographic characteristics between rural and urban AI/AN individuals in the US. Compared to rural AI/AN residents, urban AI/AN residents were younger (*p* =.045), more likely to report higher levels of education (36.6% vs. 37.2% with some college/Associate’s degree and 10.0% vs. 18.7% with a Bachelor’s degree or more, respectively; *p* =.007) and reported higher household incomes (12.8% vs. 26.1% with ratios 4.0 + times the federal poverty threshold, *p* <.001). Health insurance coverage and IHS access varied between rural and urban AI/AN adults. When examining health insurance categories stratified by IHS access, rural AI/AN individuals reported access to the IHS in greater percentages for all health insurance categories compared to urban AI/AN people (*p* <.001). For example, 24.7% of rural residents reported public insurance only with IHS compared to 1.9% of urban residents. Similarly, 9.4% of rural residents reported private insurance only and access to the IHS compared to 2.3% of urban residents, while 15.1% of rural residents reported private insurance only without IHS compared to 35.7% of urban residents. Finally, 22.4% of rural and 20.5% of urban AI/AN people were uninsured, but in rural areas, a higher percentage of uninsured people accessed IHS care; 12.8% of rural and 3.2% of urban AI/AN adults were uninsured and reported IHS access, while 9.6% of rural and 17.3% of urban AI/AN individuals were uninsured and did not report access to the IHS.

**Table 1:** Sociodemographic characteristics of AI/AN rural residents, compared with AI/AN urban residents in the united States (2019–2023)

		Rural AI/AN (*n* = 828) %	Urban AI/AN (*n* = 1,766) %	*p*-value*
**Sex**	*Female*	55.4	53.1	0.622
	*Male*	46.9	44.6	
**Age** ^a^	*18–44*	46.2	54.5	0.045*
	*45–54*	16.3	15.1	
	*55–64*	18.9	16.0	
	*65+*	18.7	14.3	
**Highest Level of Education Completed** ^b^	*Less than High School*	21.1	17.9	0.007*
	*High School Degree/Equivalent*	32.3	26.3	
	*Some college/Associate’s Degree*	36.6	37.2	
	*Bachelor’s Degree or more*	10.0	18.7	
**Disability**	*Yes*	16.2	14.4	0.286
	*No*	83.8	85.6	
**Marital Status** ^**c**^	*Married*	35.9	34.4	0.785
	*Not Married*	64.1	65.6	
**Household Income-Poverty Threshold Ratio**	*Below 1.0*	31.6	17.2	< 0.001*
	*1.0–1.99.0.99*	28.9	24.1	
	*2.0–2.99.0.99*	18.5	18.8	
	*3.0–3.99.0.99*	8.2	13.8	
	*4.0+*	12.8	26.1	
**Insurance** ^**d**^	*Private insurance only*,* with IHS*	9.4	2.3	< 0.001*
	*Private insurance only*,* without IHS*	15.1	35.7	
	*Private and Public*,* with IHS*	2.8	0.8	
	*Private and Public*,* without IHS*	4.6	6.9	
	*Public only*,* with IHS*	24.7	1.9	
	*Public only*,* without IHS*	21.0	32.0	
	*Uninsured/unknown*,* with IHS*	12.8	3.2	
	*Uninsured/unknown*,* without IHS*	9.6	17.3	

AI/AN: American Indian/Alaska Native, including those who identify as Hispanic; IHS: Indian Health Service

^a^Age category “65+” also includes less than 10 individuals with unknown age

^b^Highest Level of Education Completed category “Less than High School” also includes 19 individuals with unknown education

^c^Marital Status category “Not Married” includes 108 individuals with unknown marital status

^d^Insurance category “Uninsured/unknown, without IHS” includes 11 individuals with unknown insurance

Data are weighted to account for complex survey design, nonresponse bias, and multiple survey cycles. All P-values are the result of second-order Rao-Scott χ^2^tests with the exception of the “Insurance” P-value, which is the result of a first-order Rao-Scott χ^2^test due to negative design correction issues with the second-order test *p* < 0.05; * indicates statistical significance

Table 2 describes rural/urban differences in key health care access measures and self-rated health among AI/AN individuals. Rural AI/AN people were more likely than urban AI/AN people to report no cost-related underuse of care in the past year (75.9% vs. 65.3%, *p* <.001). Approximately half of both rural (52.4%) and urban (46.5%) AI/AN people reported not being worried about paying medical bills (*p* =.292). Rural AI/AN people were more likely than urban AI/AN people to report having a usual place for medical care (93.8% vs. 84.8%; *p* <.001). The vast majority of AI/AN adults in both rural (85.6%) and urban (89.0%) areas reported one or zero emergency visits in the past year (*p* =.071) and more than 90% in both rural (92.5%) and urban (91.1%) communities reported having had a clinic visit within the past 3 years (*p* =.463). Around three-quarters (73.8%) of rural and (78.5%) of urban AI/AN people reported excellent, very good, or good health (*p* =.015).

**Table 2:** Health care access and outcomes for AI/AN rural residents, compared with AI/AN urban residents in the united States (2019–2023)

	Rural AI/AN (*n* = 828) %	Urban AI/AN (*n* = 1,766) %	*p*-value*
**No cost-related underuse of care**,** past year**	75.9	65.3	< 0.001*
**Not worried about paying medical bills**	52.4	46.5	0.292
**Has usual place for medical care**	93.8	84.8	< 0.001*
**One or zero emergency visits**,** past year**	85.6	89.0	0.071
**Less than 3 years since last clinic visit**	92.5	91.1	0.463
**Excellent**,** very good**,** or good self-rated health**	73.8	78.5	0.015*

AI/AN: American Indian/Alaska Native, including those who identify as Hispanic. Respondents with missing values were treated as not having the variable indicated, which included 13 individuals with no response to cost-related underuse of care, 15 with no response to worry about paying medical bills, 13 with no response on usual place of care, 17 with no response on emergency visits, 13 with no response on time since last clinic visit, and less than 10 with no response on self-rated health. Data are weighted to account for complex survey design, nonresponse bias, and multiple survey cycles. All P-values are the result of second-order Rao-Scott χ^2^tests. *P*< 0.05; * indicates statistical significance

Table 3 examines differences in these same access and outcome measures by IHS access among rural and urban AI/AN residents. Those reporting IHS access were more likely than those without IHS to report no cost-related underuse of care in the past year, in both rural (86.2% vs. 65.6%, *p* <.001) and urban (76.4% vs. 63.8%, *p* =.009) areas. Reports of not being worried about paying medical bills did not differ significantly by IHS access in rural areas; approximately half of those with IHS access (53.4%) and without IHS access (51.4%) were not worried about paying medical bills. However, being worried about paying bills differed by IHS access in urban areas; urban AI/AN adults with IHS access were more likely than those who did not access IHS to say they were not worried about paying medical bills (64.0%. vs. 55.1%; *p* =.007). Having a usual place for medical care varied significantly by access to the IHS in both rural and urban areas. While the majority of AI/AN adults reported having a usual place for medical care, this was 10% points higher among AI/AN adults with IHS compared to those without IHS access in both rural (99.2% vs. 88.4%; *p* <.001) and urban (95.9% vs. 83.8%; *p* <.001) areas. The proportion of those reporting a clinic visit in the past 3 years did not differ significantly by IHS access in rural areas (93.3% of those with IHS access and 91.7% of those with no IHS access), but in urban areas, those reporting IHS access were more likely to report a clinic visit within the last 3 years than those who did not report IHS access (97.5% vs. 90.5%, *p* =.006). At least 8 in 10 rural and urban residents reported one or zero emergency visits in the past year and at least 7 in 10 reported excellent, very good, or good health, regardless of rurality and IHS access.

**Table 3:** Differences in health care access and outcomes by access to the IHS among AI/AN rural and urban residents in the united States (2019–2023)

	Rural			Urban		
	IHS Use (*n* = 372) %	No IHS Use (*n* = 456) %	*p*-value*	IHS Use (*n* = 179) %	No IHS Use (*n* = 1587) %	*p*-value*
**No cost-related underuse of care**,** past year**	86.2	65.6	< 0.001*	76.4	63.8	0.009*
**Not worried about paying medical bills**	53.4	51.4	0.779	64.0	55.1	0.007*
**Has usual place for medical care**	99.2	88.4	< 0.001*	95.9	83.8	< 0.001*
**One or zero emergency visits**,** past year**	87.9	83.3	0.206	84.5	89.4	0.223
**Less than 3 years since last clinic visit**	93.3	91.7	0.523	97.5	90.5	0.006*
**Excellent**,** good**,** or very good self-rated health**	76.0	71.6	0.277	80.8	78.3	0.461

IHS: Indian Health Service; AI/AN: American Indian/Alaska Native, including Hispanic. Respondents with missing values were treated as not having the variable indicated, which included 13 individuals with no response to cost-related underuse of care, 15 with no response to worry about paying medical bills, 13 with no response on usual place of care, 17 with no response on emergency visits, 13 with no response on time since last clinic visit, and less than 10 with no response on self-rated health. Data are weighted to account for complex survey design, nonresponse bias, and multiple survey cycles. All P-values are the result of second-order Rao-Scott χ^2^tests with the exception of the Rural “Has usual place for medical care” P-value, which is the result of a first-order Rao-Scott χ^2^test due to negative design correction issues with the second-order test.*p*< 0.05; * indicates statistical significance

## Discussion

Findings from this analysis indicate rural/urban differences in socio-demographic characteristics and some aspects of health care access and outcomes among AI/AN adults in the U.S. between 2019 and 2023. Rural-residing AI/AN adults facing greater socioeconomic risks but were also more likely to report having IHS care, as well as better access to and affordability of health care. Overall, these findings highlight high levels of excellent, very good, or good health, having a usual place of care as well as regular clinic visits, and low emergency care use among rural and urban AI/AN adults. Still, this analysis showed that more than 1 in 4 rural and 1 in 5 urban AI/AN adults reported that they are in fair or poor health, indicating potential unmet health care needs. AI/AN rural residents faced greater socio-economic risks —including lower educational attainment and income —compared to urban AI/AN adults. This may translate to higher overall health needs among AI/AN people living in rural areas, highlighting both the broad health-related challenges common in rural communities [24] and the specific issues faced in Native nations and rural Tribal communities [25]. 

Results showed that rural AI/AN adults were more likely to be able to afford needed care and be slightly less worried about paying medical bills (though that difference was not statistically significant,) compared to urban AI/AN residents. These rural/urban differences may be partially explained by the role of IHS, which was associated with greater affordability for both rural and urban AI/AN adults in this analysis. Rural AI/AN individuals were more likely to report IHS access, which reflects comparatively greater availability of ITU health care facilities in rural vs. urban settings, as well as the structure and funding of the ITU system [3]. Several independent sources indicate that the overall IHS budget is insufficient to meet patient needs, [3, 26] and this likely affects both rural and urban residents, but may be more of a detriment to urban-dwelling AI/AN adults due to currently available resource allocation largely to rural areas.

Access to a usual place of care was high overall, and AI/AN people in both rural and urban communities who accessed the IHS were more likely to have this basic health resource. Indeed, having a usual place of care was near universal among both rural (99.2%) and urban (95.9%) AI/AN adults with IHS care during 2019–2023. Additionally, over 85% of both rural and urban AI/AN adults had one or zero emergency visits over the past year during 2019–2023, and use of emergency care did not differ significantly based on whether the respondent reported IHS access. However, without access to health care records or clinical diagnoses, it is not possible to ascertain the reasons for emergency visits and draw implications about this for the different groups that were studied in this analysis.

More than 9 in 10 AI/AN adults (both rural and urban) reported having a clinic visit in the past three years. These high rates may reflect both a high level of demand for care during COVID-19 and reasonable availability of services. In both rural and urban settings, IHS access was associated with a higher percentage of respondents with a clinic visit. Notably, IHS access was correlated with a substantially higher differential (7% points) in having a clinic visit in urban AI/AN adults, compared with rural AI/AN adults (1.6% points). There are persistent and significant health challenges among some AI/AN adults in both rural and urban areas,^4^ and this study’s findings add descriptive data that suggest more research is warranted to understand unmet health needs, and whether and how access to IHS care may facilitate access to needed care among rural and urban AI/AN adults [3]. 

This study found that nearly 75% of AI/AN adults (both rural and urban) described their health as excellent, very good, or good. By comparison, around 90% of rural and urban adults generally report excellent, very good, or good health, [27] indicating that levels of good health have the potential to be higher, possibly via targeted efforts to build AI/AN health care infrastructure in both rural and urban communities [1]. Cultural and community supports and revitalization efforts are flourishing in many AI/AN communities [28]. Prior research indicates that cultural connections and community assets are important sources of social support in Tribal communities [29, 30]. Partnership with Tribal-led initiatives including language, education, ceremony, traditional healing, and workforce efforts in both rural and urban settings has the potential to increase culturally specific programming associated with positive health outcomes for AI/AN people [31, 32]. 

The United States has a federal trust responsibility to provide for the health of AI/AN individuals [33]. The ITU health system, tasked with fulfilling this responsibility, has struggled to meet patient needs [34]. This analysis showed a relationship between having IHS access and having a usual place of care and reduced cost barriers for both rural and urban AI/AN adults. However, barriers to access and usage of ITU facilities may differ between rural and urban AI/AN individuals. Further research, including analyses that are focused on particular Tribes, IHS regions, or communities, could explore the ways in which the ITU system may affect heath care access and outcomes differently for rural and urban AI/AN people.

## Limitations

There are many ways that the NHIS data, which we used for this analysis, and other federal surveys collect data that do not fully capture relevant aspects of AI/AN people and their health. This analysis was specifically limited by the fact that NHIS does not contain information on Tribal enrollment or citizenship status, residence on reservation lands, and eligibility for and access to IHS care or the other components of the ITU system. The survey questions ask about IHS in the context of health insurance, and the ITU system provides health care, not health insurance. There is no information about Tribal health facilities or urban Indian health clinics in the survey, nor is information collected on the use of Purchased/Referred Care services through IHS.

Self-assessment of AI/AN identity in a question about race does not account for tribal citizenship and sovereignty in determining membership within a community, and using AI/AN as a racial category may miscategorize people who identify as Indigenous but may be native to lands outside the borders of the United States [14, 35]. 

Additionally, the rural or urban residency of individual AI/AN respondents may change over time during the study period, as AI/AN people may move back and forth between rural and urban communities for reasons related to employment, family responsibilities, health care, ceremony, or other factors [36]. 

Attention to improving data collection and reporting practices could help provide more accurate information on the health of AI/AN rural and urban residents [37–39]. With currently available data, acknowledgement of the limitations of these data is essential [14, 35]. 

## Conclusion

The majority of AI/AN individuals in both rural and urban settings self-reported positive overall health with variability between groups in socio-economic risk factors and access to health care. Among AI/AN adults, those with access to the IHS more frequently had a usual place of care and reduced financial barriers. Results from these analyses indicate greater socio-economic health risks among rural versus urban AI/AN people. While socio-economic risks were higher for rural vs. urban AI/AN adults, health care was more accessible and affordable among rural AI/AN adults who had greater access to IHS care than urban AI/AN adults.
