# Correction: Rural/Urban Differences Among American Indian/Alaska Native Peoples in Health Care Access and Outcomes, 2019–2023

**DOI:** 10.1007/s10900-026-01571-0

**Published:** 2026-05-29

**Authors:** Katy Backes Kozhimannil, Hailey A. Baker, Ingrid E. Jacobson, Julia D. Interrante, Kyle X. Hill, Melissa L. Walls

**Affiliations:** 1 Division of Health Policy and Management, University of Minnesota School of Public Health, Minneapolis, USA; 2 Division of Environmental Health Sciences, University of Minnesota School of Public Health, Minneapolis, USA; 3 Department of International Health, Johns Hopkins University Bloomberg School of Public Health, Baltimore, USA

**Correction to: Journal of Community Health**

10.1007/s10900-025-01538-7

The author list for this manuscript was missing Ingrid E. Jacobson, MPH, who has been added. A complete list of authors and affiliations is below.

Katy Backes Kozhimannil, PhD, MPA¹; Hailey A. Baker, MD^1^; Ingrid E. Jacobson, MPH^1^; Julia D. Interrante, PhD, MPH¹; Kyle X. Hill, PhD, MPH^2^; Melissa L. Walls, PhD, MA^3^.

¹Division of Health Policy and Management, University of Minnesota School of Public Health.

^2^Division of Environmental Health Sciences, University of Minnesota School of Public Health.

^3^Department of International Health, Johns Hopkins University Bloomberg School of Public Health.

The Author Contribution Statement is as follows: All authors contributed to the study conception and design. Material preparation, data acquisition and analysis were performed by Katy Kozhimannil, Julia Interrante, and Ingrid Jacobson. The first draft of the manuscript was written by Katy Kozhimannil, and all authors commented on previous versions of the manuscript and approved the final version.

Additionally, funding sources, disclosures and acknowledgements were not included in the originally published version, and are as follows: This research was supported by the Federal Office of Rural Health Policy (FORHP), Health Resources and Services Administration, Department of Health and Human Services under Public Health Service Cooperative Agreements 5U1CRH03717. The content is solely the responsibility of the authors and does not represent the official views of the funding entities. The information, conclusions, and opinions expressed are those of the authors, and no endorsement by any funder is intended or should be inferred. FORHP provided input on the study design as part of the cooperative agreement that funded the research and confirmed that manuscript was within the scope of the cooperative agreement. The funders had no role in data acquisition, analysis, or interpretation of data. The authors gratefully acknowledge helpful input and support from Khadija Abdi, BS, and Mariana Tuttle, MPH.

